# Degradation effects of dietary solvents on microhardness and inorganic elements of computer-aided design/computer-aided manufacturing dental composites

**DOI:** 10.1186/s12903-024-03905-7

**Published:** 2024-02-01

**Authors:** Selva Malar Munusamy, Lee Ching Helen-Ng, Mohideen Salihu Farook

**Affiliations:** 1 Department of Restorative Dentistry, Level 5, Postgraduate and Research Tower, Faculty of Dentistry, University of Malaya, Kuala Lumpur, 50603 Malaysia; 2 Biomaterials Research Laboratory, Level 7, Postgraduate and Research Tower, Faculty of Dentistry, University of Malaya, Kuala Lumpur, 50603 Malaysia

**Keywords:** CAD/CAM, Composite resins, Solvents, Element, Hardness

## Abstract

**Background:**

Computer-aided design/computer-aided manufacturing (CAD/CAM) dental composites were introduced with superior mechanical properties than conventional dental composites. However, little is known on effects of dietary solvents on microhardness or inorganic elemental composition of CAD/CAM composites.

**Objectives:**

The objectives of this study were to evaluate the degradation effects of each dietary solvent on the microhardness of the different CAD/CAM dental composites and to observe the degradation effects of dietary solvent on the inorganic elements of the dental composites investigated.

**Methods:**

Fifty specimens with dimensions 12 mm x 14 mm x 1.5 mm were prepared for direct composite (Filtek Z350 XT [FZ]), indirect composite (Shofu Ceramage [CM]), and three CAD/CAM composites (Lava Ultimate [LU], Cerasmart [CS], and Vita Enamic [VE]). The specimens were randomly divided into 5 groups (*n* = 10) and conditioned for 1-week at 37^°^C in the following: air (control), distilled water, 0.02 N citric acid, 0.02 N lactic acid and 50% ethanol-water solution. Subsequently, the specimens were subjected to microhardness test (KHN) using Knoop hardness indenter. Air (control) and representative postconditioning specimens with the lowest mean KHN value for each material were analyzed using energy dispersive X-ray spectroscopy (EDX). Statistical analysis was done using one-way ANOVA and *post hoc* Bonferroni test at a significance level of *p* = 0.05.

**Results:**

Mean KHN values ranged from 39.7 ± 2.7 kg/mm^2^ for FZ conditioned in 50% ethanol-water solution to 79.2 ± 3.4 kg/mm^2^ for VE conditioned in air (control). With exception to LU, significant differences were observed between materials and dietary solvents for other dental composites investigated. EDX showed stable peaks of the inorganic elements between air (control) and representative postconditioning specimens.

**Conclusions:**

The microhardness of dental composites was significantly affected by dietary solvents, except for one CAD/CAM composite [LU]. However, no changes were observed in the inorganic elemental composition of dental composites between air (control) and 1-week postconditioning.

## Introduction

Over half a century, dental composites have evolved through innovation in manufacturing process and composition with the aim to bridge the gap in replacing mechanical, physical and aesthetic properties of lost tooth tissues [1]. Composite restorations made chairside are known as direct composites and usually consist of organic matrix as well as filler component [2]. The mechanical properties of direct composites can be improved by reducing the filler size to nanometers [3]. Contrarily, indirect composites are made extraorally either artisanal or using computer-aided design/computer-aided manufacturing (CAD/CAM) technology. The two subclasses of CAD/CAM composite blocks are dispersed fillers and polymer-infiltrated ceramic network (PICN). The dispersed fillers subclass includes Lava Ultimate (3 M ESPE, St. Paul, Minnesota, USA), Cerasmart (GC Corporation, Tokyo, Japan) and Shofu Block HC (Shofu Inc., Kyoto, Japan) that are manufactured using high temperature (> 100 °C). The only available material in the PICN subclass is the Vita Enamic (VITA Zahnfabrik, Bad Säckingen, Germany) that is manufactured using high temperature/high pressure (HT/HP) polymerization and has 86% glass-ceramic sintered network as filler composition [4, 5]. CAD/CAM composite blocks display higher material homogeneity, fewer flaws, better reliability, superior mechanical and wear properties than handmade materials [6, 7].

Microhardness is material’s hardness when subjected to low applied loads [8]. The microhardness of dental composites strongly correlates to compressive strength, degree of conversion (DC), resistance to abrasion and in vivo softening [8, 9]. Hardness measurement can be a predictor to the long-term durability of dental composites in the oral environment. The common method used for measuring hardness of polymeric materials such as dental composites is Knoop hardness (KHN) test because it minimizes the degree of elastic recovery after removal of applied load [10, 11].

Dental composites are exposed to degradation from food and beverages either intermittently or constantly [12, 13]. Based on Food and Drug Administration (FDA) guidelines, dietary solvents can be used to mimic food and beverages [14]. The degradation effects of dietary solvents on dental composites can be seen within 7 days *in-vitro* [12, 15] whereby, the highest changes in hardness had been observed within the first week of conditioning in past research [11, 16].

The high temperature and/or high pressure (HT/HP) polymerization of CAD/CAM composites contributed to the high polymer crosslink density that promoted their successful longevity in the oral environment [17]. In addition, the resistance of dental composites’ to degradation may be influenced by the characteristic features and concentration of filler particles [18]. Most research reported that the degradation of dental composites were mainly due to softening of resin matrix, changes in polymer network structure, and hydrolysis of silane couplers at the filler-matrix-interface [19–21]. Only a few studies investigated the degradation effects of dietary solvents on fillers of dental composites [22–24]. CAD/CAM restorative materials with high filler fractions had been reported to be minimally degraded in solvents [23, 24] and only one study reported the baseline elemental analysis on CAD/CAM dental composites [23].

Therefore, the objectives of this study were to evaluate the degradation effects of each dietary solvent on the microhardness of different CAD/CAM dental composites and to observe the degradation effects of dietary solvent on the inorganic elements of the dental composites investigated. The null hypothesis was that the microhardness of the different CAD/CAM dental composites would not be significantly affected after conditioning in dietary solvents.

## Materials and methods

### Specimen preparation and conditioning

One direct (Filtek Z350 XT [FZ]), one indirect (Shofu Ceramage [CM]), and three CAD/CAM dental composites (Lava Ultimate [LU], Cerasmart [CS], and Vita Enamic [VE]) of Shade A2 were investigated and their composition are displayed in Table 1. G*Power statistical software version 3.1.9.7 was used to determine the specimen size based on an analysis of variance (ANOVA) test, power: 0.80, α:0.05, effect size: 2.4 [25, 26]. A total of 10 specimens per group were prepared for each material. For FZ and CM specimens, a customized stainless steel mold with recess of 12 × 14 × 1.5 mm was used whereby, the materials were placed in one increment and compressed between two glass slides to remove excess. A light-emitting diode (LED) curing unit (Demi Plus, Kerr, Orange, CA, USA) with 8-mm-diameter light guide, output irradiance of 1330 mW/cm^2^, and 450–470 nm wavelength range was used to cure the FZ specimens with four overlapping irradiations of 20 s. The consistency of LED curing unit light output was determined using a LED radiometer (Demetron LED Radiometer, Kerr) to ensure full depth cure. A calibrated Solidilite V (Shofu Dental, Kyoto, Japan) laboratory curing unit with 4 halogen lamps (total power of 600 W; wavelength range 400-550 nm) was used to irradiate CM specimens. Subsequently, the specimens were removed from their molds following an initial cure for 1 min before a 3 min full depth cure. In order for post-cure to occur, both FZ and CM specimens were incubated at 37 degrees Celsius for 24 h. A high-speed diamond saw (Micracut 176, Metkon, Bursa, Turkey) was used to section LU, CS, and VE of 12 × 14 mm CAD/CAM blocks into 1.5 mm thickness under water coolant. All specimens were examined for defects before polished for 30 s on both sides using a twin-variable speed grinding and polisher machine (Buehler, Lake Bluff, IL, USA) in a sequence using silicon carbide abrasive paper discs from super-fine (P600) to ultra-fine (P1200) at 250 rpm [27]. After polishing, the direct, indirect, and CAD/CAM composite specimens were measured for uniformity (± 0.15 mm) using a digital micrometer (Mitutoyo Corporation, Kawasaki, Japan) before subjected to ultrasonic cleaning to remove contaminants. For each material, 50 specimens were randomly divided into five groups (*n* = 10). Each group of specimens (*n* = 10) were conditioned together for 1-week at 37 °C in a sealed petri dish containing air as control and 10 ml of the following dietary solvents: distilled water, 0.02 N citric acid (pH 2.6), 0.02 N lactic acid (pH 2.6), and 50% ethanol-water solution. The dietary solvents were not replenished during the conditioning period and after 1-week postconditioning, the specimens were rinsed with distilled water and blotted dry.

**Table 1:** Composition and manufacturers of materials investigated

Material (Abbreviation)	Manufacturer	Manufacturing process	Monomer composition	Filler composition	Filler % by weight	Lot number
Filtek Z350 XT (FZ)	3 M ESPE, St Paul, MN, USA	Direct	Bis-GMA, Bis-EMA, UDMA, TEGDMA, PEGDMA	Silica nanoparticles, zirconia nanoparticles	78.5	N771467
Shofu Ceramage (CM)	Shofu, Kyoto, Japan	Indirect	UDMA (+ HEMA in opaque paste)	Silica-based glass	74	011605
Lava Ultimate (LU)	3 M ESPE, St Paul, MN, USA	CAD/CAM	UDMA	SiO_2_ (20 nm), ZrO_2_ (4–11 nm), ZrO_2_/SiO_2_ clusters	79	N554839
Cerasmart (CS)	GC, Tokyo, Japan	CAD/CAM	UDMA + other DMA	SiO_2_ (20 nm), barium glass (300 nm)	71	1410271
Vita Enamic (VE)	VITA Zahnfabrik, Bad Säckingen, Germany	CAD/CAM	UDMA + TEGDMA	86% glass-ceramic sintered network	86	20160422

Abbreviations: Bis-EMA: ethoxylated bisphenol-A-glycidyl methacrylate; Bis-GMA: bisphenol-A glycidyl methacrylate; CAD/CAM: computer-aided design/computer-aided manufacturing; DMA: dimethacrylate; HEMA: 2-hydroxyethyl methacrylate; PEGDMA: polyethylene glycol dimethacrylate; SiO_2_: silicon dioxide; TEGDMA:triethylene glycol dimethacrylate; UDMA: urethane dimethacrylate; ZrO_2_:zirconium dioxide

### Microhardness testing and energy dispersive X-ray spectroscopy (EDX) analysis

Following conditioning, microhardness of specimens was measured using a Knoop hardness indenter (Microhardness Tester HMV-2T FA, Shimadzu, Tokyo, Japan), with 10 gf load and 15 s dwell time. For a given specimen, the three microhardness values for each surface were averaged and reported as a single Knoop hardness (KHN, kg/mm^2^) value. In order to analyze elemental composition changes, energy dispersive X-ray spectroscopy (EDX) analysis was done on air (control) and representative postconditioning specimens with the lowest mean KHN value using energy dispersive X-ray spectrometer (Oxford X-Max Instruments, Inca Software, Crest Co., Holland) of the Quanta FEG 250 scanning electron microscope (Thermo Fisher Scientific, Brno, Czech Republic). Acceleration voltage of 10 kV was used.

### Statistical analysis

Microhardness data was statistically analyzed using the SPSS Statistic 24.0 software (IBM, Chicago, IL, USA). Since data was found to be normally distributed with the Kolmogorov-Smirnov test, parametric analysis was performed. Inter-medium and inter-material differences were analyzed individually using one-way ANOVA and *post hoc* Bonferroni test at a significance level of *p* = 0.05.

## Results

Table 2 showed that the mean KHN values and standard deviations of the composite materials after conditioning in the various dietary solvents ranged from 39.7 ± 2.7 kg/mm^2^ for FZ conditioned in 50% ethanol-water solution to 79.2 ± 3.4 kg/mm^2^ for VE exposed to air (control). The inter-medium comparisons for the various materials and inter-material comparisons following conditioning in dietary solvents are shown in Tables 3 and 4, respectively. In general, VE showed the highest microhardness at air (control) and remained harder than FZ, CM, LU and CS after conditioning in various dietary solvents.

**Table 2:** Mean (standard deviation) KHN values for the various materials after conditioning

Dietary solvents	Mean KHN values (kg/mm^2^)				
	FZ	CM	LU	CS	VE
Air (control)	43.9(1.5)	46.6(2.1)	50.5(0.9)	44.2(2.7)	79.2(3.4)
Distilled water	43.7(2.9)	46.5(2.6)	50.1(2.8)	44.1(2.4)	79.1(3.5)
0.02 N Citric acid	42.5(2.3)	46.5(3.5)	49.7(0.7)	43.9(1.7)	77.5(3.5)
0.02 N Lactic acid	40.2(3.4)	46.2(1.8)	48.9(2.0)	43.5(2.3)	77.4(2.7)
50% Ethanol-water	39.7(2.7)	41.7(2.7)	49.5(2.4)	40.1(2.8)	64.7(3.2)

Abbreviations: CM: Shofu Ceramage; CS: Cerasmart; FZ: Filtek Z350 XT; LU: Lava Ultimate; VE: Vita Enamic; KHN: Knoop hardness

**Table 3:** Comparison of mean KHN values between dietary solvents based on individual material^*a*^

Materials	Differences between dietary solvents
FZ	Air (control),Distilled water > 0.02 N Lactic acid,50% Ethanol-water; 0.02 N Citric acid > 50% Ethanol-water
CM	Air (control),Distilled water,0.02 N Citric acid,0.02 N Lactic acid > 50% Ethanol-water
LU	NS
CS	Air (control),Distilled water,0.02 N Citric acid,0.02 N Lactic acid > 50% Ethanol-water
VE	Air (control),Distilled water,0.02 N Citric acid,0.02 N Lactic acid > 50% Ethanol-water

Abbreviations: CM: Shofu Ceramage; CS: Cerasmart; FZ: Filtek Z350 XT; LU: Lava Ultimate; VE: Vita Enamic; KHN: Knoop hardness; NS: no significance

^a^ Results of one-way analysis of variance and post hoc test (*p* < 0.05);

> indicates statistically significant differences in mean KHN values between conditioning in different dietary solvents for each material

**Table 4:** Comparison of mean KHN values between materials based on individual dietary solvent^*a*^

Dietary solvents	Differences between composite materials
Air (control)	VE > LU > CM,CS,FZ
Distilled water	VE > LU > CS, FZ; VE > CM
0.02 N Citric acid	VE > LU,CM,CS,FZ; LU,CM > FZ; LU > CS
0.02 N Lactic acid	VE > LU,CM,CS > FZ; LU > CS
50% Ethanol-water	VE > LU > CM,CS,FZ

Abbreviations: CM: Shofu Ceramage; CS: Cerasmart; FZ: Filtek Z350 XT; LU: Lava Ultimate; VE: Vita Enamic; KHN: Knoop hardness

^a^ Results of one-way analysis of variance and post hoc test (*p* < 0.05);

> indicates statistically significant differences in mean KHN values between different materials after conditioning in each dietary solvent

In Table 3, with the exception to LU, significant differences were observed between materials and dietary solvents for all other dental composites investigated whereby, the highest mean KHN value was observed in air (control) and the lowest mean KHN value was seen following conditioning in 50% ethanol-water solution. The significant difference in mean KHN values between dietary solvents for CM, CS and VE were ranked alike. For FZ, significant difference in mean KHN value was observed between conditioning in citric acid and 50% ethanol-water solution, but not with lactic acid.

The mean KHN value of VE was significantly higher compared with other dental composites when conditioned in all the dietary solvents (Table 4). Significant difference in mean KHN values were ranked alike when conditioned in air (control) and 50% ethanol-water solution. Almost similar pattern to the former was observed when conditioned in distilled water, with exception to CM that was significantly less hard than VE. Conditioning in citric acid showed that VE was significantly harder than other composites tested, whereas LU and CM were significantly harder than FZ only. CS and LU presented similar results after conditioning in citric acid and lactic acid, whereby LU was significantly harder than CS. No significant differences in mean KHN were observed between FZ and CS in all dietary solvents except when conditioned in lactic acid, whereby CS was significantly harder than FZ.

**Fig. 1:**
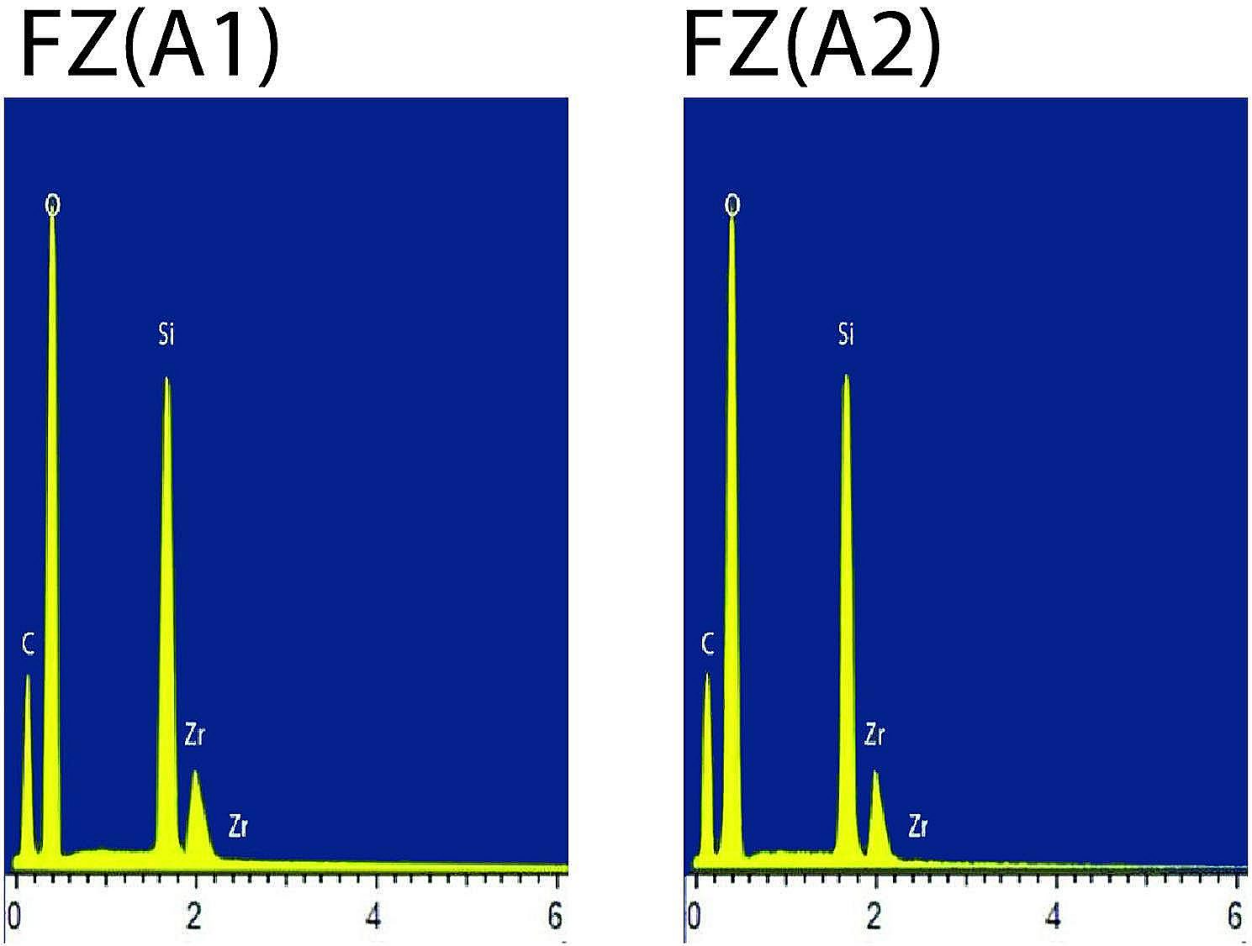
EDX spectra of Filtek Z350 XT for air (control) [FZ(A1)] and after conditioning in 50% ethanol-water [FZ(A2)]

**Fig. 2:**
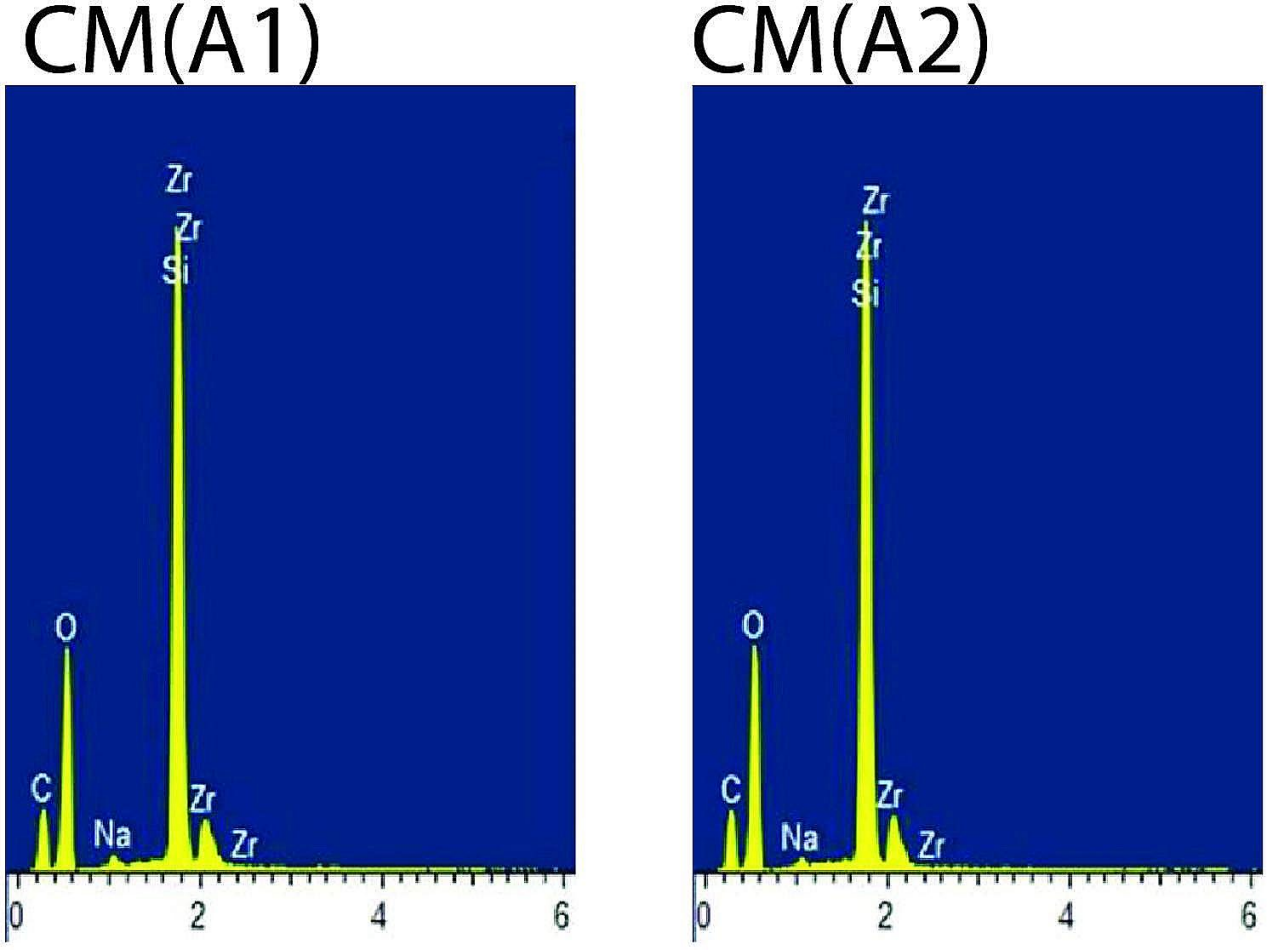
EDX spectra of Ceramage for air (control) [CM(A1)] and after conditioning in 50% ethanol-water [CM(A2)]

**Fig. 3:**
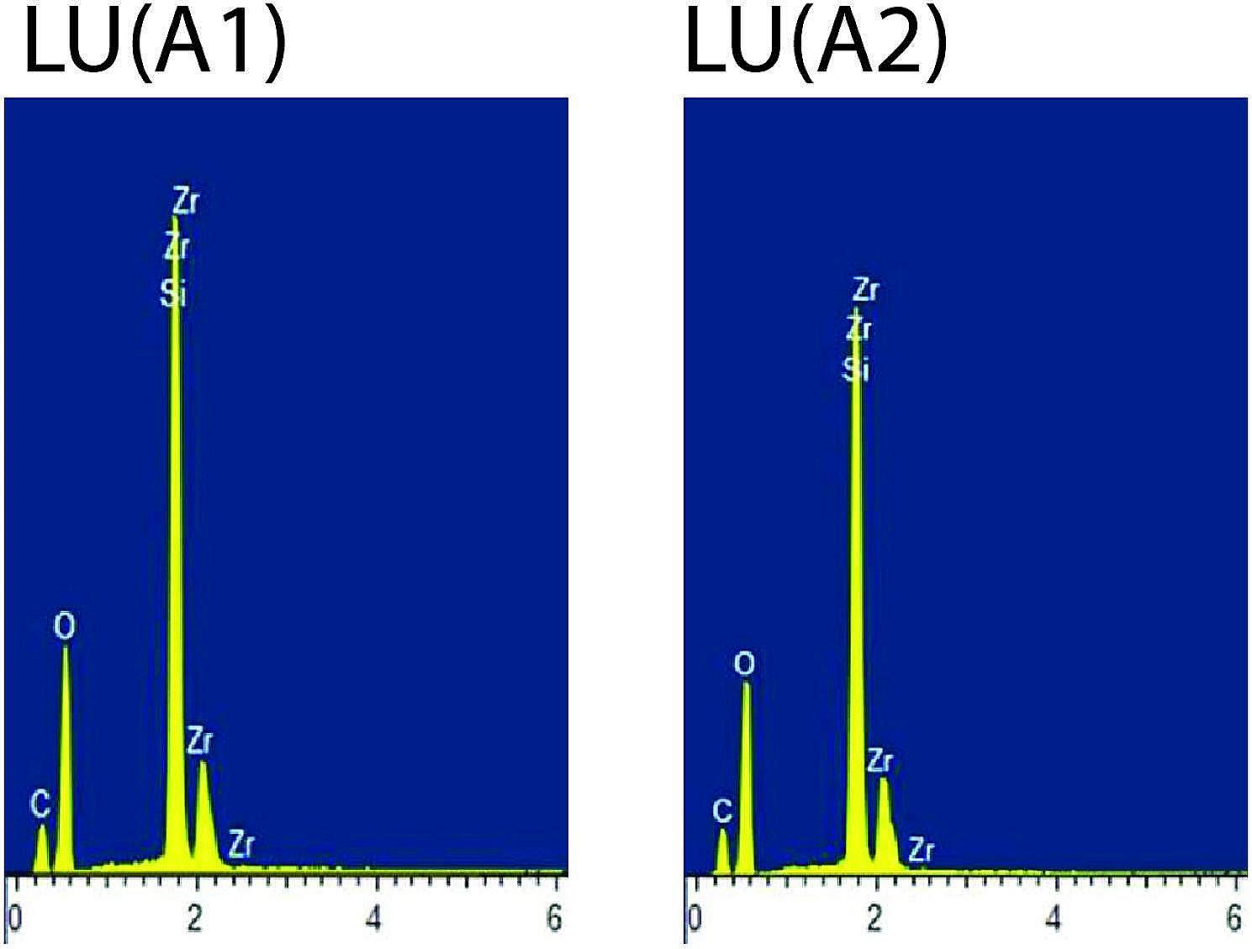
EDX spectra of Lava Ultimate for air (control) [LU(A1)] and after conditioning in 0.02 N lactic acid [LU(A2)]

**Fig. 4:**
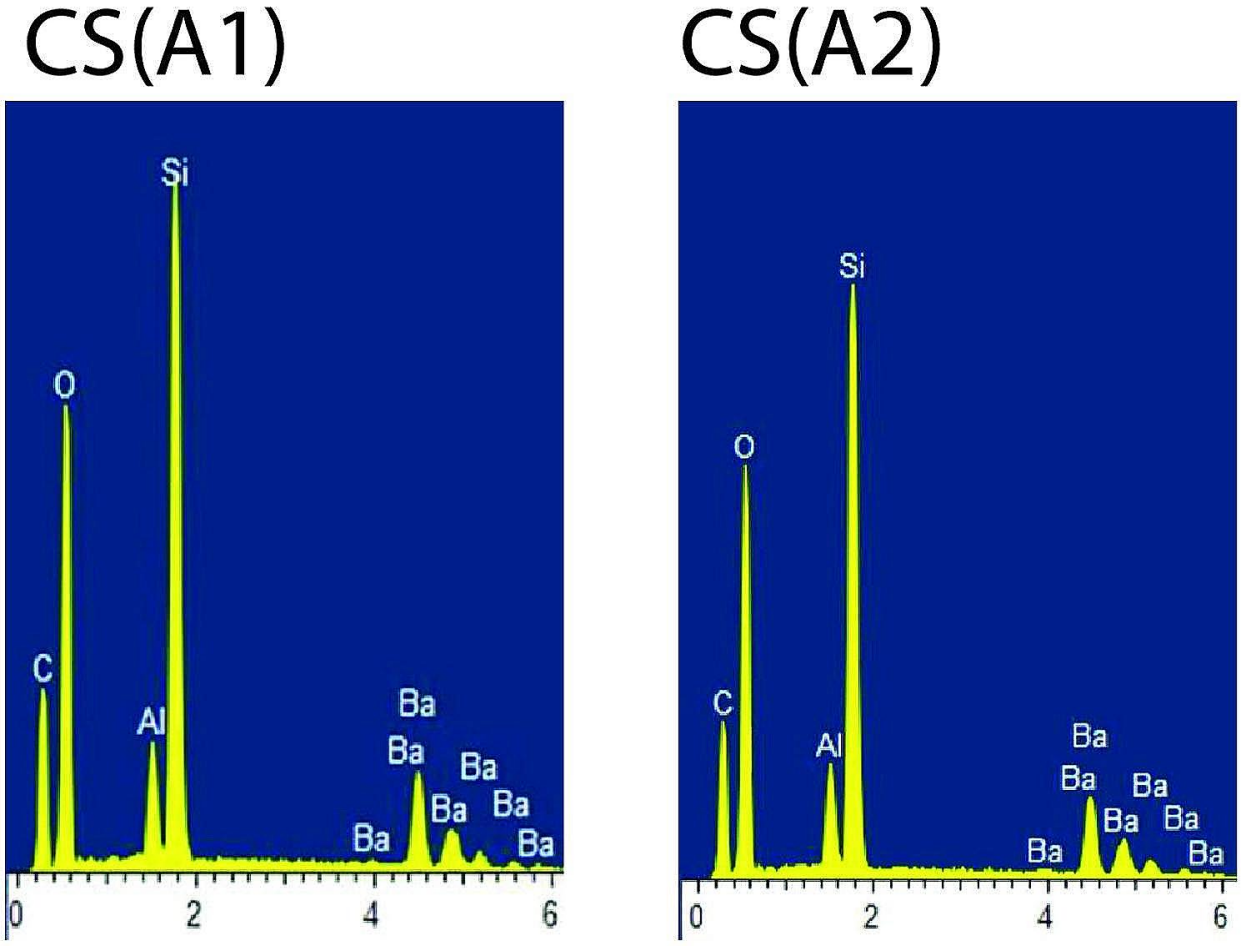
EDX spectra of Cerasmart for air (control) [CS(A1)] and after conditioning in 50% ethanol-water [CS(A2)]

**Fig. 5:**
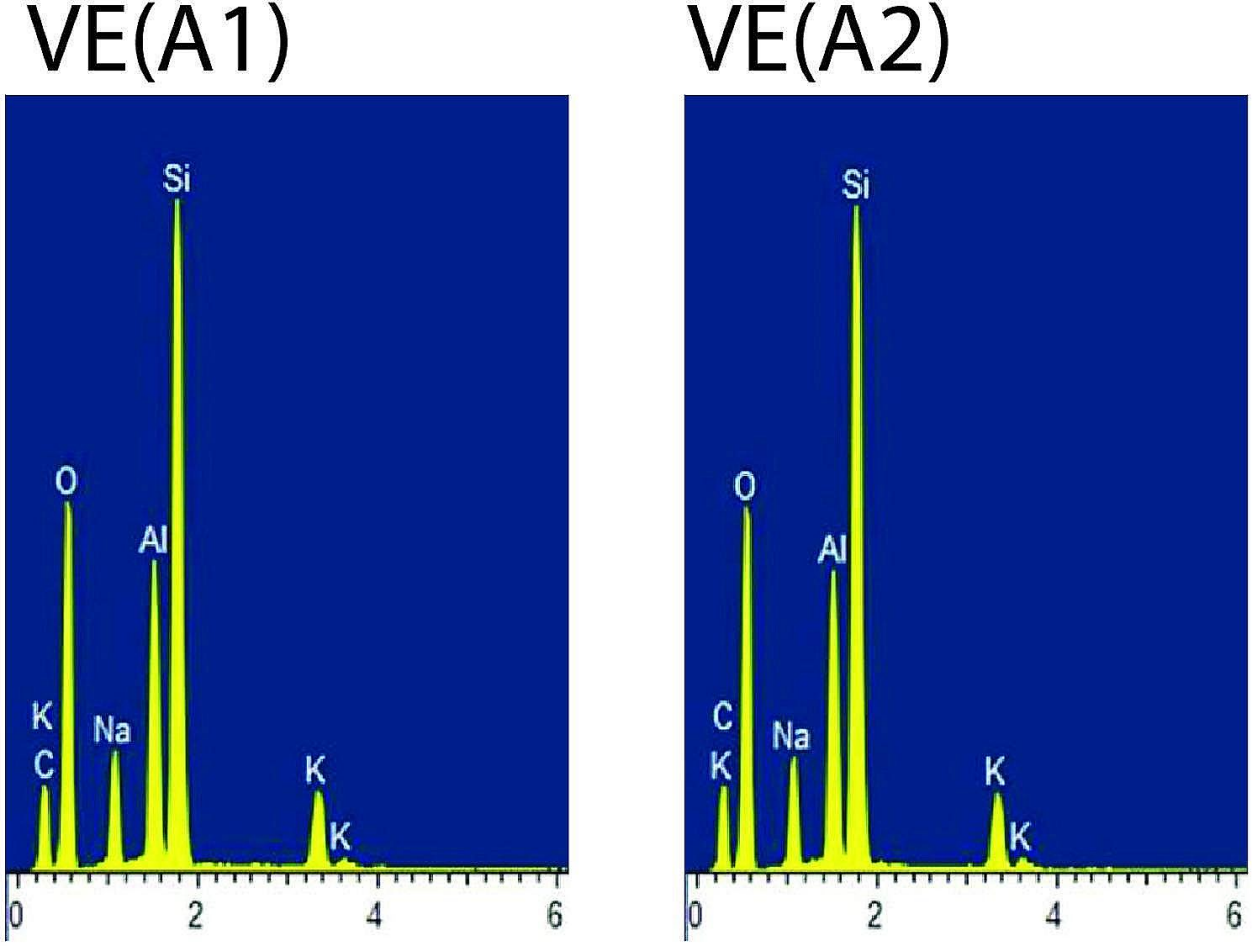
EDX spectra of Vita Enamic for control [VE(A1)] and after conditioning in 50% ethanol-water [VE(A2)]

For each material, one specimen conditioned in air (control) and one representative postconditioning specimen with the lowest mean KHN value was subjected to Energy Dispersive X-ray Spectroscopy (EDX) analysis. Overall, the organic elements such as C (carbon) and O (oxygen) were present abundantly in all types of composite materials. FZ showed stable EDX spectra peaks of the inorganic elements such as Si (silicon) and Zr (zirconia) for air (control) and after conditioning in 50% ethanol-water (Fig. 1).CM showed stable EDX spectra peaks of the inorganic elements such as Si (silicon), Zr (zirconia) and Na (sodium) for air (control) and after conditioning in 50% ethanol-water (Fig. 2). LU displayed stable EDX spectra peaks of the inorganic elements such as Si (silicon) and Zr (zirconia) for air (control) and after conditioning in 0.02 N lactic acid (Fig. 3). CS displayed stable EDX spectra peaks of the inorganic elements such as Si (silicon), Al (aluminium) and Ba (barium) for air (control) and after conditioning in 50% ethanol-water (Fig. 4), whereas VE showed stable EDX spectra peaks of the inorganic elements such as Si (silicon), Al (aluminium), Na (sodium) and K (potassium) for air (control) and after conditioning in 50% ethanol-water (Fig. 5).

The element quantification in percentage by Energy Dispersive X-ray Spectroscopy (EDX) for air (control) and postconditioning representative specimen with lowest mean KHN value for each material was shown in Table 5. The difference in the inorganic element percentage and atomic weight ratios between air (control) and postconditioning representative specimens was minimal with ± 1% respectively.

**Table 5:** Element quantification in percentage by energy dispersive X-ray spectroscopy (EDX) for air (control) and postconditioning representative materials with lowest mean KHN value for each material

Material	Element in Weight%															
	Air (control)								Postconditioning							
	C	O	Si	Zr	Na	Al	Ba	K	C	O	Si	Zr	Na	Al	Ba	K
FZ	24.51	40.68	21.24	13.57	-	-	-	-	23.32	40.82	21.86	14.00	-	-	-	-
CM	27.55	42.74	22.85	6.26	0.60	-	-	-	27.82	42.58	22.86	6.40	0.34	-	-	-
LU	22.51	43.07	22.08	12.34	-	-	-	-	26.76	39.72	20.72	12.80	-	-	-	-
CS	29.74	39.44	15.33	-	-	2.34	13.15	-	31.95	37.50	15.84	-	-	2.77	11.94	-
VE	18.78	44.93	20.21	7.86	4.60	-	-	3.62	19.14	45.01	20.09	7.81	4.38	-	-	3.57

Abbreviations: Al: Aluminum; Ba: Barium; C: Carbon; CM: Shofu Ceramage; CS: Cerasmart; FZ: Filtek Z350 XT; K: Potassium; KHN: Knoop hardness; LU: Lava Ultimate; Na: Sodium; O: Oxygen; Si: Silicon; VE: Vita Enamic; Zr: Zirconia

## Discussion

The microhardness of direct, indirect and two out of three CAD/CAM dental composites investigated were significantly affected after conditioning in dietary solvents. LU was the only CAD/CAM dental composite not significantly affected despite showing significant differences in the mean KHN values between composite types postconditioning in dietary solvents. Therefore, the null hypothesis was rejected. EDX results showed no changes in the inorganic elemental composition between air (control) and representative postconditioning specimens with the lowest mean KHN value for each material investigated.

Microhardness can be an important indicator to the degree of conversion (DC) of dental composites whereby, microhardness of dental composites correlates with their mechanical properties [19]. However, the mechanical properties of dental composites are strongly influenced by the monomer system, filler loading, filler size and filler-resin interface [28]. In the present study, a range of dental composites that undergo different manufacturing process were selected in order to evaluate the degradation effect of dietary solvents on their differences. Generally, the CAD/CAM dental composites investigated had higher KHN values at air (control) than the direct and indirect composite with exception to CS. The KHN value for CS at air (control) was only slightly above FZ (direct composite) but lower than CM (indirect composite). This could be due to the manufacturing process that may have improved the DC of CS to fare better than FZ but since CS displayed the lowest filler % by weight compared to all materials investigated, the KHN value for CS at air (control) was still lower than CM.

Water sorption into dental composites can affect the mechanical properties by causing hydrolytic breakdown of filler-silane bond, filler-matrix debonding or hydrolytic degradation of fillers [29]. The rate of water sorption is influenced by DC and chemistry of monomers in dental composites [30]. Although the highest DC was found in TEGDMA compared to other monomers such as Bis-GMA, Bis-EMA and UDMA, TEGDMA can cause an increase in water sorption and curing shrinkage, thus negatively affecting the properties of matrix resin [31]. In present study, postconditioning in distilled water displayed the least reduction on microhardness values of all materials investigated and this is in agreement with previous finding [32]. LU showed superior stability with no significance differences when conditioned in air (control), distilled water, 0.02 N citric acid and 0.02 N lactic acid as compared with CM, CS and VE. The possible explanation to this finding is that LU contained only UDMA and is categorized as a homopolymer that has been shown to display high DC and homogeneity when polymerized using HT/HP technology [33]. Since CM, CS and VE were composed of UDMA and other methacrylates, they fall under the category of copolymers that may affect their stability in dietary solvents. Contrarily, FZ (direct composite) showed no significant differences in postconditioning mean KHN values between air (control) and distilled water. FZ which is also categorized as a copolymer was the only material investigated that had Bis-GMA in the monomer composition. Combination of Bis-GMA/TEGDMA has been shown to display synergistic effect on the polymerization rate of dental composites compared to the mixture of UDMA/TEGDMA or Bis-EMA/TEGDMA [31]. The resistance of FZ to water sorption may be increased by the blend of copolymers and the improved packing of spherical shaped nanoparticles that reduce the fillers’ stress raising effect [20]. However, FZ was still found to be inferior to withstand acid attack as compared to the indirect and CAD/CAM composites investigated.

Solvents can reduce hardness and soften polymers by creating a plasticizer molecule that acts as a space occupier to separate polymer chains and lower the effectiveness of entanglements [30, 34]. Solubility parameter of dental composites is directly related to the matrix resin whereby the highest softening effect is observed when the solubility parameter of a solvent and the matrix polymer of the composite is equal [35]. In the current study, the materials investigated showed the highest decrease in microhardness after conditioning in alcohol group compared to distilled water or acid groups and this is in agreement with previous finding [34]. 50% ethanol-water solution can cause softening of Bis-GMA based dental composites [35]. Most dental monomers (Bis-GMA, Bis-EMA, UDMA, D_3_MA) absorbed higher amount of pure ethanol than water or ethanol-water solution except for TEGDMA, that absorbed more amounts of ethanol-water solution than water or pure ethanol [30, 36]. The aforementioned may explain the stability of LU in 50% ethanol-water solution and other dietary solvents investigated since it was composed of only UDMA. Although VE had the highest filler content than all materials investigated, significant reduction in microhardness following postconditioning in 50% ethanol-water solution was observed. Interestingly, this was in contrast to recent research that reported little changes in hardness of CAD/CAM dental composites with high filler fractions following 7-day storage in 75% ethanol-water solution [24]. The filler composition (glass-ceramic sintered network) in VE that differed from the other composites investigated may have attributed to the findings of the current study. Furthermore, VE is a copolymer and the presence of TEGDMA compared to only UDMA in LU can cause an increase in water sorption, thus cause a significant reduction in microhardness of VE following postconditioning in 50% ethanol-water solution.

The mild to severe negative effect of acids on microhardness of dental composite depends on the material composition, solvent type and immersion time [32]. Softening of Bis-GMA based polymers by acids was very distinct when the polymer is composed of copolymer with diluting monomer such as TEGDMA [37]. Apart from FZ (direct composite), the indirect and CAD/CAM composites showed no statistically significant differences between acids and distilled water. The aforementioned finding on CAD/CAM composites is in accordance with a recent study that demonstrated similar effect on hardness value of CAD/CAM resin composites after aging 7-days in acidic drink and demineralized water [24]. FZ was the only material in our study that had Bis-GMA with TEGDMA as a diluting monomer that may cause it to be vulnerable to softening effect of acids. The type of acid may affect pH and the pH influence on microhardness of dental composites has been reported to be material dependent [38]. This corroborates with our results where the postconditioning mean KHN values for FZ showed no significance difference between 0.02 N lactic acid and 50%ethanol-water solution but not with 0.02 N citric acid.

Silicon was the major constituent in the filler composition of the materials tested in this study. Since EDX results showed stable peaks in the inorganic elemental composition of CAD/CAM composites following postconditioning in dietary solvents, it is possible that leaching of inorganic ions from fillers did not contribute to the reduction of microhardness of the materials in the 1-week conditioning period. Instead, dietary solvents may have degraded the dental composites through softening of resin matrix after 1-week conditioning whereby, significant reduction in microhardness was observed in materials composed of copolymer. This corroborates with past study where leaching of inorganic ions was reported to start late due to the diffusion-controlled process and leaching was seen more prominent in exposed fillers than from fillers covered in resin and silane [29]. Filler composition and filler treatment were responsible for the diversity in leaching of inorganic ions and the total amount of leachable ions corresponded to the DC of polymer network of dental composites [39, 40]. Although recent research reported that when conditioned in solvents, minimal degradation was observed in CAD/CAM restorative materials with high filler fractions [23, 24], the findings of this study showed that the filler composition and high filler fraction of CAD/CAM dental composites may synergistically influence their resistance to degradation effects of dietary solvents on microhardness.

Most CAD/CAM dental composites were found to be susceptible to degradation effects of dietary solvents despite high temperature and/or high pressure (HT/HP) polymerization except for LU. From this study, it can be suggested that the combination of homopolymer in particular UDMA and dispersed fillers with high fraction in CAD/CAM dental composites may increase their resistance to degradation effects of dietary solvents on microhardness. A longer conditioning period in dietary solvents is recommended to investigate the leaching of fillers in dental composites whereby, an atomic absorption spectrophotometry can be done to determine the concentration of leached inorganic ions from dental composites into dietary solvents. Apart from that, the degree of conversion can be investigated using FTIR to identify the effect of dietary solvents on the chemical functional groups and molecular structures in both organic and inorganic compounds. In addition, the use of artificial saliva as a solvent can be considered since it closely represents the oral environment and may affect leaching of inorganic ions from dental composites.

## Conclusions

Within the limitation of our study, it can be concluded that the microhardness (KHN) of dental composites was significantly affected except for one CAD/CAM dental composite, LU and no changes were observed in the inorganic elemental composition of CAD/CAM dental composites 1-week postconditioning in dietary solvents.

## Data Availability

The data used and/or analyzed during the current study are available from the corresponding author upon reasonable request.
